# New Molecular Mechanisms and Markers in Inflammatory Disorders, 2nd Edition

**DOI:** 10.3390/ijms26136346

**Published:** 2025-07-01

**Authors:** Emanuela Rita Galliera, Elena Vianello

**Affiliations:** 1 Department of Biomedical Sciences for Health, Università Degli Studi di Milano, 20122 Milan, Italy; emanuela.galliera@unimi.it; 2 IRCCS Ospedale Galeazzi-Sant’Ambrogio, 20157 Milan, Italy; 3 Experimental Laboratory for Research on Organ Damage Biomarkers, IRCCS Istituto Auxologico Italiano, 20133 Milano, Italy

This article belongs to the Special Issue “New Molecular Mechanisms and Markers in Inflammatory Disorders, 2nd Edition”.

Similar to the first edition (link: https://www.mdpi.com/journal/ijms/special_issues/Mechanisms_Inflammatory (accessed on 26 June 2025)), this second edition of *New Molecular Mechanisms and Markers in Inflammatory Disorders* focuses on deepening our understanding of the origins of inflammatory diseases, highlighting the most advanced knowledge related to major inflammatory disorders.

In recent years, significant progress has been made in understanding the molecular underpinnings of inflammatory states. These conditions encompass a range of pathological disorders, from rheumatic diseases to autoimmune responses, from tumor onset to neurodegenerative states—all characterized by complex immune responses and chronic tissue inflammation. As previously highlighted, identifying molecular mechanisms and biomarkers is critical not only for early diagnosis and prognosis but also for the development of targeted therapies.

In this issue, readers will find nine contributions, which may be accessed here: https://www.mdpi.com/journal/ijms/special_issues/K876690MOQ (URL access on 26 June 2025) that they are summarized in Figure 1.

Wozniak et al. explored the impact of biological markers that were useful for classifying patients admitted to the emergency department (ED) and stratifying them based on mortality risk and health deterioration. They reported the crucial relevance of the soluble urokinase plasminogen activator receptor (suPAR) as a promising serological predictor of mortality risk for ED patients (Contribution 1).

Among chronic disorders, Tarasovs et al. demonstrated the complexity of osteoarthritis (OA)—a chronic condition that affects the joints and impairs mobility—by revealing the interconnection between inflammation, pain, and cognitive decline. They reported that urinary TNF-α and TGF-β1 may serve as biomarkers reflecting inflammation and disease severity in OA, suggesting that synovial inflammation may be linked to mental and cognitive health in certain patient cohorts (Contribution 2).

Equally important, Baek et al. contributed a paper to this Special Issue, focusing on one of the most prevalent disorders in developed countries: metabolic obesity. In this context, they conducted a cross-sectional study analyzing the association between metabolic obesity phenotypes and inflammatory markers in a specific cohort of Korean adults. Their findings revealed varying profiles of systemic inflammation across different metabolic obesity phenotypes, using high-sensitivity C-reactive protein (hs-CRP) as a key marker (Contribution 3).

In a related study, Bahman et al. elucidated the molecular mechanisms underlying the overexpression of IL-6 in adipose tissue associated with obesity. They reported that a TNF-α/stearate cooperative model drives IL-6 expression in 3T3-L1 cells via an H3K9/18Ac-dependent mechanism, with implications for the exacerbation of adipose IL-6 in obesity (Contribution 4).

To further explore the link between inflammation and insulin resistance (IR), Mirabelli et al. presented a narrative review discussing the role of hypoxia in obesity. They outlined the most recent insights into the interplay between hypoxia, inflammation, and metabolic dysregulation, which is central to the pathogenesis of obesity-related IR (Contribution 5).

A major development in the field of biomarkers has been the discovery of non-coding RNAs, such as microRNAs (miRNAs) and long non-coding RNAs (lncRNAs), which regulate gene expression post-transcriptionally and are increasingly recognized as key modulators of immune cell differentiation and function. In this context, Escolar-Peña et al. identified a specific microRNA profile associated with inflammation and lipid metabolism in patients with allergic asthma. They discovered four miRNAs (hsa-miR-99b-5p, hsa-miR-451a, hsa-miR-326, and hsa-miR-505-3p) as potential biomarkers for stratifying allergic asthmatic patients by severity and for providing insights into the molecular pathways of severe uncontrolled asthma (Contribution 6).

No less important, the immunological perspective is fundamental to understanding the inflammatory pathways of these disorders. Hernandez-Suarez et al. investigated whether the presence of reactive antibodies against membrane antigens in tissues from both animal models and humans could serve as biomarkers in autoimmune disorders. They concluded that specific IgG antibodies against membrane antigens in patients’ serum may be of great relevance for both clinical and basic research as potential biomarkers of immune dysfunction (Contribution 7).

Inflammatory disorders of acquired origin are also of growing interest, particularly those related to alcohol abuse. Niemelä et al. reported that patients with alcohol use disorders (AUDs) exhibit IgA immune responses to ethanol metabolites and tissue transglutaminase. These immune responses coincide with the activation of inflammation, extracellular matrix remodeling, and the generation of aberrantly glycosylated proteins (Contribution 8).

Finally, this Special Issue features a narrative review that focuses on the molecular mechanisms of viral infections. Sun et al. highlighted the pivotal role of mitochondrial function and damage induced by viral infections, as well as their involvement in pro-inflammatory and innate immune responses. They proposed mitochondria as potential therapeutic targets for various infectious diseases (Contribution 9).

We sincerely thank all the authors who submitted their work and contributed to this collection, as well as the staff of the *International Journal of Molecular Sciences* for their invaluable support in making this editorial project a success. All articles are freely available as open access and distributed under the terms of the Creative Commons Attribution (CC BY) license 4.0 (https://creativecommons.org/licenses/by/4.0/ (accessed on 26 June 2025)).

## Figures and Tables

**Figure 1:**
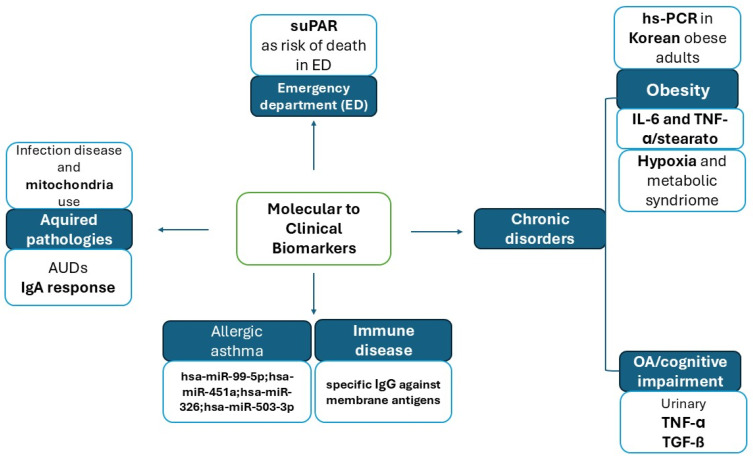
This figure summarizes the topics in this Special Edition. Authors propose different molecular biomarkers for inflammatory disorders through their work, aiming to bring them into clinical practice. We collect nine papers focused on five major scientific points: Emergency intervention, chronic disorders, allergic and immune disorders, and acquired pathologies. The authors proposed different biomarkers for each of them, useful in understanding the complexity of these disorders.

## Abbreviation

Soluble urokinase plasminogen activator receptor (suPAR), high-sensitivity C-reactive protein (hs-CRP), osteoarthritis (OA), and alcohol use disorders (AUDs).

